# The New Anesthetics

**Published:** 1899-09

**Authors:** 


The New Anesthetics.—Our foreign exchanges contain very favorable reports of the reception of the new anesthetics, orthoform, "orthoform neu" and nervanin. The dentists are especially enthusiastic. One mentions that he extracted twenty-two teeth with nervanin at one sitting (vide *Journal*, p. 311). Bardet combines ethyl chlorid with 2 to 4 percent cocain or eucain and applies it as a spray or tampon, thus avoiding the prick of the syringe. It is especially effective for application on the mucous membranes, gums, anns, etc. The tests with it are described in the *Presse Med.* of January 24th.

